# Fingertip injuries

**DOI:** 10.4103/0019-5413.32051

**Published:** 2007

**Authors:** Sanjay Saraf, VK Tiwari

**Affiliations:** Department of Burns, Plastic and Maxillofacial Surgery, Safdarjung Hospital, New Delhi, India

**Keywords:** Avulsed fingertip, fingertip injuries, fingertip lacerations

## Abstract

**Background:**

Fingertip injuries are extremely common. Out of the various available reconstructive options, one needs to select an option which achieves a painless fingertip with durable and sensate skin cover. The present analysis was conducted to evaluate the management and outcome of fingertip injuries.

**Materials and Methods:**

This is a retrospective study of 150 cases of fingertip Injuries of patients aged six to 65 years managed over a period of two years. Various reconstructive options were considered for the fingertip lesions greater than or equal to 1 cm^2^. The total duration of treatment varied from two to six weeks with follow-up from two months to one year.

**Results:**

The results showed preservation of finger length and contour, retention of sensation and healing without significant complication.

**Conclusion:**

The treatment needs to be individualized and all possible techniques of reconstruction must be known to achieve optimal recovery.

The hand is prone to domestic and industrial trauma with fingertips being the most frequently injured portion of the hand.^1^ Fingertip resurfacing is a challenging reconstructive problem as the treatment varies widely and is thus controversial. The goals of treatment are to maintain the length of the digit as well as to provide well padded, stable and sensate yet pain-free skin. We retrospectively reviewed the management and outcome of 150 cases of fingertip injuries managed over a period of two years.

## MATERIALS AND METHODS

A retrospective analysis of 150 cases of fingertip injuries treated by different methods over a period of two years was undertaken. Fingertip injuries were defined as lesions greater than or equal to 1cm^2^ in the terminal phalanx. A detailed history including patient's demographics, mechanism of injury, hand dominance, occupation, duration since injury and tetanus immunization status was taken. The male predominance (M: F 90:60) was seen due to increased exposure and occupational hazards. The majority of the males were either agricultural or industrial workers. The age group involved was six to 65 years. The pediatric cases (n = 14) predominantly were due to door crush injury. The females (n = 34) predominantly had kitchen or household injuries. The males (n = 102) were predominantly victims of work-related mishaps, the majority being agricultural and industrial-related accidents. Crush injury (n = 48) was found to be the commonest cause of fingertip trauma, followed by laceration (n = 30) and avulsion injuries (n = 16).

The injuries were evaluated in a careful and systematic manner for finger involvement, crush versus sharp injuries, location, depth, angle of the defect, nail bed involvement and status of the remaining soft tissue, co-morbid conditions and the configuration of the fingertip defect. Standardized radiographs and photographs were also taken.

**Figure 1:**
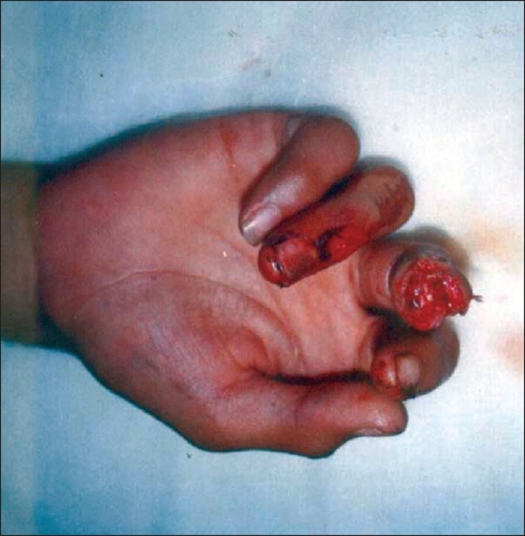
Transverse fingertip defect

**Figure 2:**
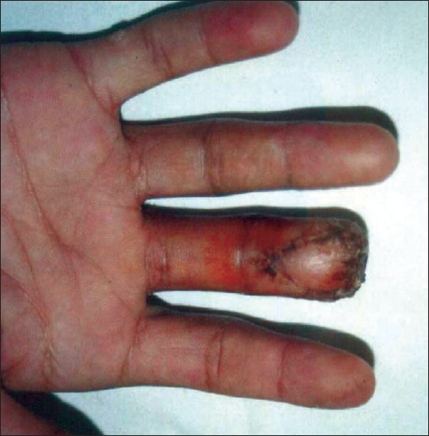
After V-Y plasty (4 weeks postoperative)

**Figure 3:**
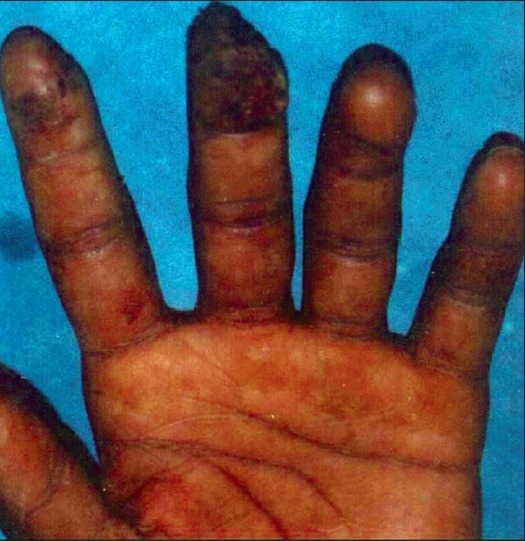
Volarly directed fingertip defect

**Figure 4:**
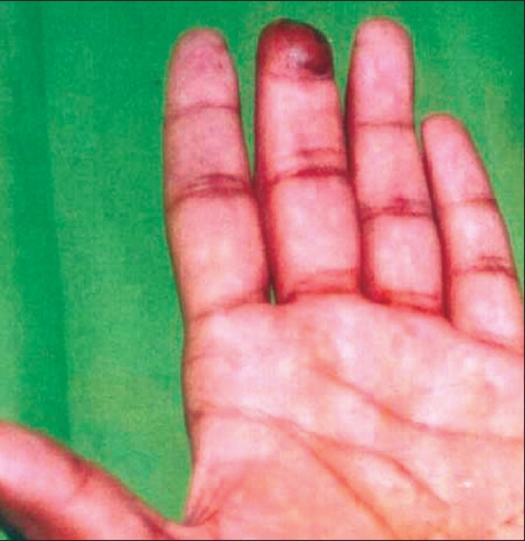
Cross-finger flap (3 months postoperative)

Various reconstructive options were considered based on the philosophy and techniques of earlier and contemporary surgeons [Table 1]. In volarly directed wounds larger than 1cm without exposed bone or tendon, split-thickness grafting (n = 20) was performed. Full-thickness grafting (n = 8) was preferred in skilled professionals like artists, computer professionals and technicians. Composite tip grafting (n = 6) was usually considered in tip amputations in children below six to seven years of age. However, in one adult girl who was a computer professional it was attempted and was successful. When bone or tendon was found to be exposed, a local flap was considered. The choice of flap was technically dictated by the extent and obliquity of the tip loss. Volar V-Y flaps (n = 25) were preferred in transverse amputations beyond the mid-nail level and dorsal oblique amputations beyond the proximal nail level [Figures 1 and 2]. Lateral V-Y flaps (n = 21) were considered for the wounds with volar and transverse avulsions with exposed bone with excess lateral skin. In the volarly directed wounds without sufficient pulp, cross-finger flap (n = 19) was preferred [Figures 3 and 4]. In oblique amputations, Venkataswami oblique flap was considered (n = 10). The thenar flap (n = 7) was preferred in females for transverse, volar and dorsal injuries involving the index and middle fingers. In elderly patients, patients with co-morbid conditions, and in unskilled laborers revision amputation was often considered.

**Table 1:** Reconstructive options considered, complications seen and results achieved

Procedure	No. of cases	Complications seen	Results
Grafting			
SSG*	20	Partial loss of graft (4)	Good
FTSG†	8	Total loss (1)	Fair
Composite graft	6	Total loss (2)	Fair
Local flaps			
Volar V-Y (Kleinert)	25	Marginal necrosis (4), nail bed pull (1). Cold intolerance (3), wound infection (1)	Good
Lateral V-Y (Kutler)	21	Marginal necrosis (3), Cold intolerance (2), wound infection (1)	Good
Venkataswami oblique flap	10	Marginal necrosis (2)	Good
Dorso V-Y	3	Dififculty in mobilization (1)	Poor
Moeberg's flap	9	Marginal necrosis (1) Nail bed pull (3), Cold intolerance (1)	Good
Regional flaps			
Cross finger flap	19	Superficial flap necrosis (2), Partial wound dehiscence(3), Partial wound detachment (3)	Fair
Thenar flap	7	Finger stiffness (3)	Fair
Reverse vascular pedicle flap	2	Hyperaesthesia (1)	Fair
Dorsal transposition flap	2	Inadequate coverage (1)	Poor
“Visor”	1	Difficulty in transposition (1)	Fair
Distant flaps			
Littler flap	6	Hyperaesthesis (2), Cold intolerance (1)	Good
Groin flap	6	Bulky (6)	Poor
Others (Revision amputation)	5		
Total	150		

* SSG - Split- thickness Skin Graft

† FTSG - Full - thickness Skin Graft

**Figure 5:**
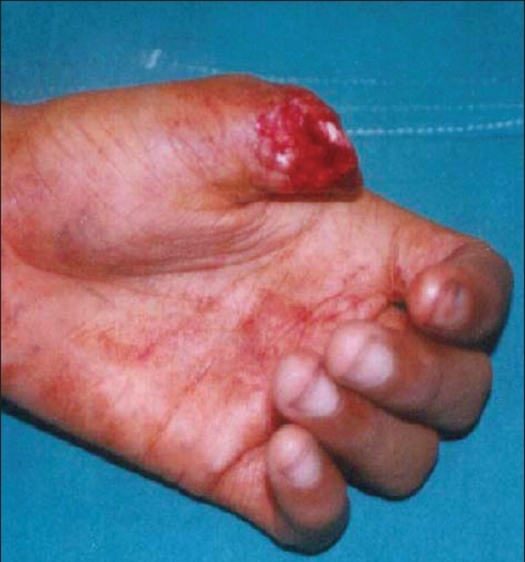
Zone II amputation thumb

**Figure 6:**
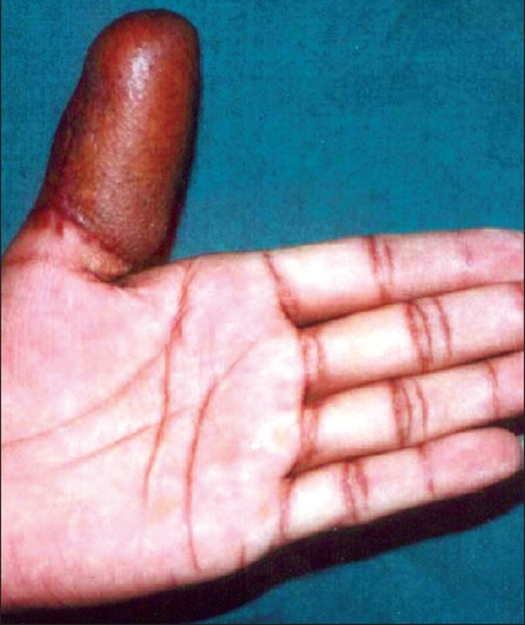
Groin flap (6 months postoperative)

In thumb tip defects less then 1.5 cm, the Moberg flap (n = 9) was preferred. In defects exceeding more then 1.5 cm first dorsal metacarpal artery flap/Littler flap/groin flap [Figures 5 and 6] were primarily done. All the nail bed lacerations were repaired under loup magnification with 6–0/7–0 Vicryl. The nail was always reposited back as a splint to reduce the subsequent nail deformities.

The total duration of treatment varied from two to six weeks. The patients were followed up from two months to one year. In follow-up, fingertips were reassessed and evaluated for length, padding, nail deformity, fingertip sensation and range of motion, and were also photographed in standardized views.

## RESULTS

Crush injury (n = 48) was found to be the commonest type of fingertip trauma, followed by lacerated (n = 30) and avulsion injuries (n = 16). The clean cut amputations (n = 18), Blunt cut amputations (n = 25) and Mangling type of injuries (n = 13) accounted for the remaining fingertip injuries. Middle finger (n = 38) followed by multiple finger injuries (n = 34) were found to be the most commonly involved. The thumb was found to be involved in 21 cases. The index (n = 29), ring (n = 18) and little finger (n = 10) accounted for the remaining injuries. The majority of the injuries occurred at work. The majority of these injuries healed with excellent results in terms of maintenance of maximum finger length and minimization of cosmetic deformity and functional disability [Table 1].

Postoperative follow-up was two months to one year. The observations were recorded for appearance, patient satisfaction, two-point discrimination, hypersensitivity and cold intolerance, numbness, pain, active range of motion and active use. The evaluation was done for general appearance, use, sensations and static two-point discrimination employing 1 2 3 grading. The results were classified as good (10), fair (5–10) and poor (< 5) depending upon the total aggregate. For static two-point discrimination the scoring criteria was: 6mm or more: 1, 3 to 6 mm: 2 and 3 mm or less: 3. All the patients were found to achieve measurable average of 6mm two-point discrimination. Nearly all the patients were satisfied with the functional result and the aesthetic contour. The incisions healed with inconspicuous scars. The work incapacity time averaged between four to eight weeks and most patients could return to their routine.

All flaps healed uneventfully, except for 10 patients in whom marginal necrosis of the flap occurred, which was managed conservatively. Partial wound dehiscence was observed in three patients. Partial wound detachment and infection was seen in three and two patients respectively. Cold intolerance was observed in seven and paresthesia in three patients. Joint stiffness was noted in three of the cases [Table 1].

## DISCUSSION

Fingertip injuries are extremely common and comprise the most common hand injuries. They are often viewed as a relatively minor injury but their improper management can lead to considerable loss of skilled hand function. Fingertip injuries lead to significant morbidity affecting the occupational as well social activities. They account for approximately 10% of all accidents reported in the casualty and two-thirds of hand injuries in children.

The management of fingertip injuries is complex and not without controversy as a variety of treatment options are available. Goals of treatment in fingertip injuries include preservation of useful sensation, maximizing functional length, preventing joint contractures, providing satisfactory appearance and avoiding donor disfigurement and functional loss.^2^

The approach to the management of fingertip injuries depends on many variables, including patient age, sex, hand dominance, profession, hobbies, finger involvement, location, depth, angle of the defect, nail bed involvement, status of the remaining soft tissue, co-morbid conditions and the anatomy of the fingertip defect.^3^ As the primary goal of treatment of an injury to the fingertip is a painless fingertip with durable and sensate skin, the knowledge of fingertip anatomy and the available techniques of treatment are of paramount interest.

Fingertip injuries can be classified according to site of the amputation and whether the injury primarily involves the pulp or nail bed and refer to the zone and the plane of injury.^3^,^4^ The injuries classified as Zone I occur distal to the distal phalanx with preservation of the majority of the nail bed and the matrix. Treatment of Zone I injuries is usually conservative.

Injuries classified as Zone II are located distal to the lunula of the nail bed and are characterized by the exposure of the distal phalanx. These injuries require flap for reconstruction.^3^ The plane of Zone II can be further classified as dorsal, transverse or volar, according to the plane of the amputation. The slope of transection and the condition of the local tissue determine the best reconstructive technique.

Injuries classified as Zone III involve the nail matrix and result in the loss of the entire nail bed. Injuries in Zone III are not considered for elaborate reconstruction.

Evans and Bernadis (2000) proposed a new PNB (pulp, nail, bone) classification system for fingertip injuries.^5^ This system classifies a fingertip injury into three areas: pulp, nail and the bone. As this classification system is new, more long-term studies will be required for its usefulness.

As fingertip injuries can be treated in different ways their management needs to be carefully individualized. If there is no or minimal tissue loss, the wound can be closed primarily with or without debridement. Healing by secondary intention or open technique by combination of wound contraction and re-epithelialization is applicable to small volarly directed fingertip wounds with no exposure of bone.^6^–^8^ This is not preferred for wounds greater than 1 cm as it takes a long time to heal with the loss of volume. This approach has a definite place for fingertip injuries in children as they have good capacity of regeneration. If the wound is larger than 1 cm and volarly directed, without exposure of bone or tendon, skin grafting provides faster healing. Split-thickness grafts are favored as contraction results in a smaller defect. However, some authors favor full-thickness grafts as they re-innervate early and provide durable coverage.^9^–^11^ Composite tip grafts are often considered for young children below the age of six years but are not reliable for adults.^12^,^13^ When bone or tendon is exposed at the base of a fingertip wound, the use of skin grafts is not feasible and a local flap is necessary.^3^

The type of flap reconstruction which is appropriate depends on the extent and configuration of the tip loss. In those amputations which are oblique, the direction and degree of obliquity also influences the choice of flaps. Local flaps if properly applied can provide a very satisfactory functional and esthetic result. The various local flaps used to reconstruct fingertips include volar V-Y, bilateral V-Y flaps, cross-finger flap, thenar flap and island flaps. Flap choice depends on the orientation and configuration of the wound, injured digit and sex of the patient. If the wound is small and involves a finger with a transverse amputation beyond the mid-nail level and dorsal oblique amputations beyond the proximal nail fold, the volar V-Y flap (Atasoy) gives good results.^14^ Bilateral V-Y (Kutler) flaps are best applied to volar and transverse avulsions with exposed bone when excess lateral skin is present.^15^ The cross-finger flap is preferable if the wound is volar-directed without sufficient volar pulp to facilitate V-Y flap. However, if local flap is not possible, a regional flap like thenar, cross-finger flap or neurovascular island flap may have to be considered.^16^–^18^ The thenar flap can be used for volar, transverse and dorsal injuries, specially for index and long fingers and is often preferred in females as it does not scar the visible dorsum.

The Venkataswami oblique triangular flap is very useful in the cases of oblique amputations.^19^ Other flaps like Reverse digital flap, Visor, Dorsal V-Y and Dorsal transposition flap may be considered in selected cases.^20^–^22^ Often microsurgical replantation for complete amputation of the tip of a digit is feasible but most often the amputated part is either not available or is badly damaged.^23^ In case of relative contraindications like advanced age, osteoarthritis or other systemic co-morbid conditions, revision amputations are preferred.

Thumb tip defects need special consideration as preservation of thumb length is always a priority for optimal hand function. The rectangular volar advancement (Moberg) is the preferred option for smaller defects less then 1.5 cm as it brings sensate durable skin to the thumb tip.^24^ In thumb defects more then 1.5 cm first dorsal metacarpal artery flap or the Littler flap are often required for glabrous and sensate resurfacing with preservation of thumb length.^25^ Large thumb defects are often best reconstructed with a free sensate flap from the great toe/first web space.^26^ Occasionally, a cross-finger flap from the dorsum of the index finger is required if the Littler flap and first dorsal metacarpal artery flap are not available for sensate resurfacing of the thumb. As the dorsal vascular anatomy is dependent on the proper digital vessels in digits, the Moberg flap should not be used in the digits.

Nail bed lacerations should be repaired preferably under loupe magnification to prevent nail plate abnormalities.^27^ Occasionally, large defects of the nail bed require split-thickness graft from an uninjured area of nail bed or from the second toe. Occasionally, in some fingertip injuries revision amputation is preferable to allow tension-free closure of the soft tissues and adequate padding in an effort to minimize recovery time and hasten return to work.

The common complications encountered postoperatively were marginal necrosis, cold intolerance and hypersensitivity. The marginal necrosis was attributable to tension closure, and other minor complications like partial wound dehiscence, partial graft loss were independent of the surgical technique employed to treat them. Hypersensitivity and cold intolerance are basically complications of the injury and not the treatment. Review of the literature suggests that the rates of hypersensitivity and cold intolerance approximate 50% regardless of the modality of treatment. However, this is self-limited and resolves within one to two years.

## CONCLUSION

The critical evaluation of fingertip defect and various techniques is necessary to choose the best possible reconstructive option from esthetic and functional recovery [Table 2]. Though the strict guidelines regarding management are difficult to formulate, the following recommendations are likely to be helpful in achieving a satisfactory functional and aesthetic result. In volarly directed wounds larger than 1cm without exposed bone or tendon, split-thickness graft is preferable. Full-thickness grafting may be preferred in skilled professionals. In children below six to seven years, composite tip grafting should be considered. If there is no or minimal tissue loss, the wound can be closed primarily. Conservative management may be employed for fingertip injuries less than 1 cm with no bone exposure.

**Table 2:** Type of defect and preferred option

Type of defect	Satisfactory result achieved
Zone I defects in children	SSG
Zone I defects in adults	SSG*/FTSG†/V-Y
In children below 6 years with total clean cut amputation, Zone II	Composite graft
Zone II defects with dorsal angulations	Volar V-Y (Kleinert)
Zone II defects with palmar angulations	Cross Finger Flap
Zone II defects with transverse amputation / slightly volar amputation	Lateral V-Y (Kutler)
Zone II defects with oblique amputation	Venkataswami Oblique triang. Flap
Zone II Thumb defects less than 1.5 cm	Moeberg's flap / Cross Finger Flap
Zone II Thumb defects more than 1.5 cm	Littler/ Cross Finger Flap
Zone III	Revision amputation/Groin flap

* SSG - Split- thickness Skin Graft

† FTSG - Full - thickness Skin Graft

The flap should be considered whenever bone or tendon is found to be exposed. The technical choice of flap is to be dictated by the anatomy of the tip loss. The volar V-Y flaps may be preferred in dorsally angulated amputations beyond the proximal nail level and transverse amputations beyond the mid-nail level. Bilateral (Lateral) V-Y flaps may be considered for the wounds with slightly volar and transverse avulsions with exposed bone with excess lateral skin. In the volarly directed wounds without sufficient pulp, cross-finger flap may be done. In obliquely directed amputations, oblique triangular flaps give satisfactory results. In females with transverse, volar and dorsal injuries, especially involving the index and middle fingers thenar flap may be preferred. In elderly patients, mentally unstable patients, osteoarthritic patients, uncontrolled diabetics and in unskilled laborers revision amputations can be considered.

In thumb tip defects less then 1.5 cm, the Moberg flap is preferable. In defects exceeding 1.5 cm first dorsal metacarpal artery flap/Littler flap may be considered. If the Littler and first dorsal metacarpal artery flap is not possible, a cross-finger flap from the dorsum of the index finger may be considered. Specialized flaps like the oblique triangular flap, reverse digital flap, dorsal metacarpal and Littler flap should only be considered if technical expertise is available.

After tip reconstructive surgery, splintage of the involved finger for 2-3 weeks should be considered for early and safe recovery. All nail bed lacerations need to be meticulously repaired using 6–0/7–0 absorbable sutures under loupe magnification. If the nail is found to be removed, it should be replaced as a splint over the repaired nail bed. If warranted, technically challenging cases should be referred to specialized hand units.
